# Literary Notes

**Published:** 1902-12

**Authors:** 


### LITERARY NOTES.

THE Charles Roome-Parmele Company, of New York, has issued a brochure, entitled, Diabetes Mellitus; its Treatment and Cure. The pamphlet contains clinical reports by some fifteen well-known physicians residing in various parts of the country and who have obtained satisfactory results—in some instances remarkable cures—from the use of arsenauro in diabetes mellitus. These reports have been published by the authors in the medical journals—*The New York Medical Journal*, *The International Medical Magazine*, and others—and they have been grouped in pamphlet form for the information of physicians. This makes them conveniently accessible and in doing this the publishers have rendered a service of value to the profession. The book will be sent on application to Charles Roome Parmele Company, 45 John Street, New York.

THE Maltine Company, Borough of Brooklyn, New York City, has just published a brochure, entitled, Neoferrum, the New Iron. The book contains a chemical history of the new agent, followed by clinical reports upon its use by Dr. Thomas M. Acken, of New York, made in the West Side German Dispensary and in private practice. The pamphlet is handsomely printed and will be supplied upon application to the Maltine Company.

THE Buffalo State Hospital has lately issued its thirty-first annual report, which is for the year ending September 30, 1901. It is an interesting pamphlet of sixty-eight pages, illustrated with numerous half-tone plates, giving views of the exterior and interior of the hospital. It appears that during the year 2,293 patients were treated, and that the minimum number under care was 1,859. It is surprising that such efficiency of administration can be obtained with this enormous number of insane patients, as Dr. Hurd and his staff are well known to have reached. The management of this hospital is a model in medical and business methods and is a credit to Buffalo as well as the state.

---

Mariani and Company, of New York and Paris, are publishing a handsome periodical called *Mariani's Coca Leaf*, the first number of which bears the date November, 1902. Its object, as stated in its foreword, is particularly to present to the medical profession the most recent information relating to the therapeutical uses of coca. It being an occasional review, its appearance will be comparatively infrequent, perhaps once a quarter, and will be of interest, it is thought, to physicians. At all events, its first number is full of information about this interesting and useful plant that should be read by even the most fastidiously and ultra ethical medical man, who sniffs commercialism in every publication that carries an advertisement of any manufactured preparation not in the pharmacopeia. This little magazine will be sent to physicians, if they will send their addresses to Mariani & Co., 52 W. 15th Street, New York.

---

The *Wichita Medical Journal* is the name of a new magazine published at Wichita, Kan., edited by Dr. G. K. Purves. Its subscription price is one dollar a year. The October number contains a number of interesting and well written articles relating to a variety of medical subjects.

